# Effects of fertilizer practice on fungal and actinobacterial cellulolytic community with different humified particle-size fractions in double-cropping field

**DOI:** 10.1038/s41598-021-97975-0

**Published:** 2021-09-16

**Authors:** Haiming Tang, Chao Li, Yilan Xu, Kaikai Cheng, Lihong Shi, Li Wen, Weiyan Li, Xiaoping Xiao

**Affiliations:** 1 Hunan Soil and Fertilizer Institute, Changsha, 410125 China; 2 Hunan Biological and Electromechanical Polytechnic, Changsha, 410127 China

**Keywords:** Biotechnology, Microbiology

## Abstract

Cellulose plays an important role in maintaining or improving soil carbon (C) cycling and soil fertility of paddy field. There had close relationship between functional cellulose genes (*cbhI* and *GH48*) with characterize of soil organic matter chemical components (fulvic acid and humic acid) and soil physical fractions. However, there is still limited information about how functional cellulose degradation response to long-term fertilizer management and their relative importance for C sequestration under the double-cropping rice paddy field in southern of China. Therefore, the objective of this study were investigated the effects of 34-years long-term fertilizer regime on community abundance of *cbhI* and *GH48* genes in five soil particle-size fractions (> 2000 μm, 2000–200 μm, 200–50 μm, 50–2 μm and 2–0.1 μm) by using polarization magic angle spinning ^13^C nuclear magnetic resonance spectroscopy. The field experiment was included four different fertilizer treatments: chemical fertilizer alone (MF), rice straw and chemical fertilizer (RF), 30% organic manure and 70% chemical fertilizer (OM), and without fertilizer input as a control (CK). The results showed that distribution of soil humus and cellulolytic microbial community abundance was significant increased under long-term application of crop residue and organic manure condition. And the FA, HA and HM C contents in > 2000 μm and 2000–50 μm fractions with MF, RF and OM treatments were significant higher than that of CK treatment. Meanwhile, the alkyl C and Oalkyl C groups of FA and HA in > 2000 μm fraction with MF, RF, OM and CK treatments were higher than that of the other fractions. There had higher AL% and lower ARO% of FA and HA in different particle-size fractions with MF, RF, OM and CK treatments. The results indicated that abundance of *cbhI* and *GH48* genes in different particle-size fractions with RF and OM treatments were significant increased, compared with CK treatment. There had significant positive correlation between soil humus C components (FA and HA) with abundance of *cbhI* and *GH48* genes, and the o-alkyl C and AL% of FA were positively correlated with abundance of *cbhI* and *GH48* genes. As a result, the community abundance of *cbhI* and *GH48* genes were significant increased under combined application of crop residue and organic manure with chemical fertilizer condition.

## Introduction

Soil organic matter (SOM) is a general term for series of organic compounds formed by various organic substances entering the soil under the action of soil microorganism, it plays a vital role in maintaining or improving soil quality and productivity^1^. Furthermore, agricultural soil is the main source of greenhouse gas emission, which is benefit for mitigating global climate warming by increased carbon (C) sequestration in agricultural soil and decreased carbon dioxide (CO_2_) emission into atmospheric^2^. Some results indicated that higher SOM content can improve soil quality and represent a substantial contribution to reduction of CO_2_ emission via C sequestration^3^. Therefore, it is beneficial practice for maintaining or improving soil quality and productivity by increased SOM content.

SOM content and quality were close related with different particle-size fractionation of soil. Particle-size fractionation, which allows for the separation of SOM pool with changing degrees of microbial alteration and mineral association, might help elucidate microbial-mediated soil C cycling characteristic^4,5^. In the previous studies, these results indicated that SOM were mostly derived from the decomposition of animal and plant litter, soil humus component were main affected by environmental change, such as soil type^6^, crop system^7^, fertilizer regime^8^ and so on. Soil humus is a complex organic matter, which the animal and crop residue were synthesized by soil microbial decomposition and transformation. Soil humus are main including of fulvic acid (FA), humic acid (HA) and humin (HM), which are the most ubiquitous non-living natural organic compounds in the soil^9^. However, there is still limited information about how the soil particle-size fractions respond to chemical and spectroscopic characteristics of soil humus under long-term fertilization condition.

Soil humification is the main process of soil C cycling with microbial-mediated, and cellulose decomposition is the critical process of soil C transformation for that cellulose is the most abundant polysaccharide in plant litter that enters soil^10^. In the previous studies, the results indicated that decomposition of cellulose was main completed through the synergistic activity of three major groups of soil enzyme, such as endoglucanases, cellobiohydrolases and β-glucosidases^11^. During the rate-limited step of cellulose decomposition, cellulolytic enzyme were main encoded by fungal glycoside hydrolase family 7 cellobiohydrolase I gene (*cbhI*) and bacterial glycoside hydrolase family 48 (*GH48*)^12^. In recent years, some results indicated that fungal *cbhI* in *Ascomycota* and *Basidiomycota* could representative group of cellulolytic fungi^13,14^, and the abundance and diversity of *GH48* gene were detected from *actinobacteria* could representative ecological role in soil C cycling^15^. Therefore, it was benefit for explanting of C transformation mechanism to investigate of the key functional gene corresponding to specific soil microorganism involved in cellulose decomposition under long-term fertilization condition^16,17^.

Rice (*Oryza sativa* L.) is one of the main crops in Asia, and double-cropping rice system (early rice and late rice) is the main land use in southern of China^18^. It is benefit practice for maintaining or improving quality and fertility of paddy field by combined application of organic fertilizer with inorganic fertilizer^2,18^. Soil physical and chemical characteristics were profound changed by taken different fertilizer management, such as pH, soil bulk density, soil organic carbon (SOC) content^18^, which in return affecting functional humus fractions and C sequestration in paddy field. In the previous studies, these results indicated that soil functional humus fractions and SOC content were usually regarded as a vital index for the change of soil quality and fertility^1,9^, and soil fungal and actinobacterial cellulolytic community plays an important role in processing of C transformation in paddy filed^11,15^. However, there is still need further to study the effects of different fertilizer management on fungal and actinobacterial cellulolytic community in different humified particle-size fractions in the double-cropping rice paddy field under long-term fertilization condition. We hypothesized that: (i) chemical and structural characteristics of soil humus would be changed under long-term fertilization condition; (ii) fungal and actinobacterial cellulolytic community abundance would be influenced by fertilization, particle-size fraction and their interaction effects, and (iii) correlation between cellulolytic microbial abundance with C content of soil humus were also changed under long-term fertilization condition. Therefore, the 34-years long-term field experiment with different fertilizer treatments were conducted in a double-cropping rice system in the southern of China. Hence, the objective of this study was: (1) to illustrate the chemical and structural characteristics of soil humus (FA, HA and HM) in different fertilizer practice under a double-cropping rice system; (2) to investigate the abundance of *cbhI* and *GH48* genes in five particle-size fractions (> 2000 μm, 2000–200 μm, 200–50 μm, 50–2 μm and 2–0.1 μm) in paddy field under long-term fertilization condition.

## Materials and methods

### Sites and cropping system

The experiment was begun in 1986. It was located in NingXiang County (28°07′ N, 112°18′ E) of Hunan Province, China. The field experiment under a continental monsoon climate, the annual mean precipitation is 1553 mm and potential evapotranspiration is 1354 mm. The monthly mean temperature is 17.2 C. At beginning of this field experiment, the soil chemical characteristics at plough layer (0–20 cm) of paddy field were as follows: soil organic carbon (SOC) 29.4 g kg^−1^, total nitrogen (N) 2.0 g kg^−1^, available N 144.1 mg kg^−1^, total phosphorous (P) 0.59 g kg^−1^, available P 12.87 mg kg^−1^, total potassium (K) 20.6 g kg^−1^, and available K 33.0 mg kg^−1^. The crop rotation system and other more detail information about the field experiment were described as by Tang et al. (2018)^19^.

## Experimental design

The field experiment was included four fertilizer treatments: chemical fertilizer alone (MF), rice straw and chemical fertilizer (RF), 30% organic manure and 70% chemical fertilizer (OM), and without fertilizer input as a control (CK). A randomized block design was adopted in the plots, with three replications of each treatment. And each plot size was 66.7 m^2^ (10.0 × 6.67 m). The field experiment were ensured that same total amount of N, phosphorus pentoxide (P_2_O_5_), potassium oxide (K_2_O) for MF, RF and OM treatments during early rice and late rice whole growth period, respectively. During the early rice and late rice whole growth period, the total amount of N, P_2_O_5_, K_2_O for MF, RF and OM treatments were 142.5, 54.0, 63.0 kg ha^−1^ and 157.5, 43.2, 81.0 kg ha^−1^, respectively. And the kind of organic manure for OM treatment was decomposed chicken manure. The kinds of chemical fertilizer were included urea, ordinary superphosphate, and potassium chloride, respectively. Before transplanting of rice seedling, air-dried rice straw were manually spread onto the soil surface and incorporated into the soil at a cultivation depth of 20 cm. During early rice and late rice whole growth period, 70% and 60% of N were applied at tillage before transplanting of rice seedling, respectively, and the remaining N were applied at top dressing stage of rice. All the P_2_O_5_ and K_2_O fertilizer were applied at tillage before transplanting of rice seedling. One-month-old of early rice and late rice seedling were transplanted with the density of 150,000 plants ha^−1^ in paddy field. Other more detail information about fertilizer management and filed arrangement were described as by Tang et al. (2018)^19^.

### Soil sampling and samples preparation

There had ensured that permission to collect soil sampling in this experiment. Undisturbed soil samples were collected from each plot in 25 August, 2019, at the tillering stage of late rice. Three soil cores (10 × 10 × 15 cm) at the depth of 0–20 cm from each plot were collected and equally merged as representative soil samples for one replicate of each fertilizer treatment. Moist soil were gently broken apart along the natural breakpoints and passed through a 5-mm sieve to remove visible organic debris and crop root. After thorough mixing, different particle-size fractions were separated according to the method described as by Stemmer et al.^20^. In the present study, five particle-size fractions were obtained for each soil samples, such as > 2000 μm, 2000–200 μm, 200–50 μm, 50–2 μm and 2–0.1 μm. These particle-size fractions were then stored at room temperature for analysis of soil chemical characteristic, at 4 °C for soil extracellular enzyme analysis and − 80 °C for the molecular analysis. The SOC, total nitrogen (TN) contents and soil enzyme activity (β-glucosidase, β-cellobiohydrolase) of the soil samples were investigated according to the method described as by Zhang et al.^21^.

### Soil laboratory analysis

#### Extraction and purification of soil humus

The extraction of different humus fractions from soil samples were performed with 5 g dry soil and 50 mL of 0.1 mol L^−1^ NaOH in 0.1 mol L^−1^ sodium pyrophosphate under a N_2_ atmosphere, and it were repeated several times until colorless supernatants were obtained. The suspensions were centrifuged at 5000 × *g* for 15 min and the pooled alkali extract were acidified to pH 2.0 with H_2_SO_4_, and kept for 24 h at room temperature. The soluble fulvic acid (FA) was separated from coagulation (humic acid (HA) fraction) by centrifugation^9^. The residue, which was the precipitate in the centrifuge tube, was collected to provide humin (HM). Total carbon (C) content of FA and HA were investigated by using multi B/C 3100 TOC/TN and C content of HM were measured by using a vario macro cube element analyzer (Elementar Analysensysteme GmbH, Hanau, Germany). The soil C content of humus (FA, HA and HM) of the soil samples were investigated according to the method described as by Jindog et al.^8^.

#### CPMAS solid-state ^13^CNMR spectroscopy

In the present study, chemical composition of purified FA and HA fractions of soil samples with MF, RF, OM and CK treatments were via CPMAS solid-state ^13^CNMR spectroscopy and expressed as the relative abundance of the major C type. The solid-state ^13^CNMR spectra were measured on a Bruker Avance III 400 NMR spectrometer (Germany) conducting at a spinning speed of 5 kHz and a contact time of 1 ms, with a ^1^H 90 °C pulse length of 4 μs and a recycle delay of 0.8 s. The chemical shift regions 0–45, 45–95, 95–165 and 165–200 ppm were referred to alkyl C, *o*-alkyl C, aromatic C and carboxylic C, respectively ^21^. The areas of the spectral regions were measured though the integration routine of the spectrometer and expressed as percentage of the sum of all spectral areas, respectively^22^. The degree of aromaticity (ARO%) and aliphaticity (AL%) of the FA and HA C were calculated according to the equations as following:

1 $$ {\text{ARO}}\left( \% \right) = \frac{{{\text{Aromatic C }}\left( {{95} - {\text{165 ppm}}} \right)}}{{{\text{C}}\;{\text{signal }}\left( {0 - {\text{165 ppm}}} \right)}} $$

2 $$ {\text{AL}}\left( \% \right) \, = \frac{{{\text{Aliphatic C }}\left( {0 - {\text{95 ppm}}} \right)}}{{{\text{C}}\;{\text{signal }}\left( {0 - {\text{165 ppm}}} \right)}} $$

The alkyl C/*o*-alkyl C ratio were calculated and regarded as an indicator of the degree of organic matter decomposition^23^.

#### Deoxyribonucleic acid (DNA) extraction and quantitative polymerase chain reaction (qPCR) assay

Soil DNA was extracted from 0.5 g fresh soil by using the FastDNA SPIN Kit (MP Biomedicals, Illkirch, France) and a Fast Prep-24 Homogenization System (MP Biomedicals, Irvine, CA) according to the manufacturer's instructions. Successful DNA extraction was characterized by using electrophoresis on 1% (wt/vol) agarose gels. The quantity and quality of DNA were checked by using Nanodrop spectrophotometer (Nanodrop, PeqLab, Germany).

Genes encoding fungal glycoside hydrolase family 7 cellobiohydrolase I gene (*cbhI*) and bacterial glycoside hydrolase family 48 (*GH48*) were selected as biomarkers of cellulolytic fungi and actinobacteria, respectively. All qPCR assays were carried out in an iCycler system (Bio-Rad, USA) by using SYBR Green I chemistry and the data were analyzed by using Bio-Rad iQ5 v2.0, quantitative PCR were investigated according to the method described as by Fan et al.^24^.

### Statistical analysis

The statistical analyses of each investigate items in this manuscript were conducted by using SAS 9.3 software package^25^. The data of each particle-size fractions with all fertilizer treatments in this manuscript were compared by using Fisher's significant difference at the *p* < 0.05 probability level. Pearson's correlation analyses were performed to assess the linear correlation among soil properties, enzyme activity, C groups of FA and HA with abundance of *cbhI* and *GH48* genes. The results of each investigate items were expressed as mean and standard error.

### Statement on guidelines

There was not used live plant in the present experimental research and field study, and comply with relevant institutional, national, and international guidelines and legislation.

## Results

### Carbon content of humus composition

**Figure 1:**
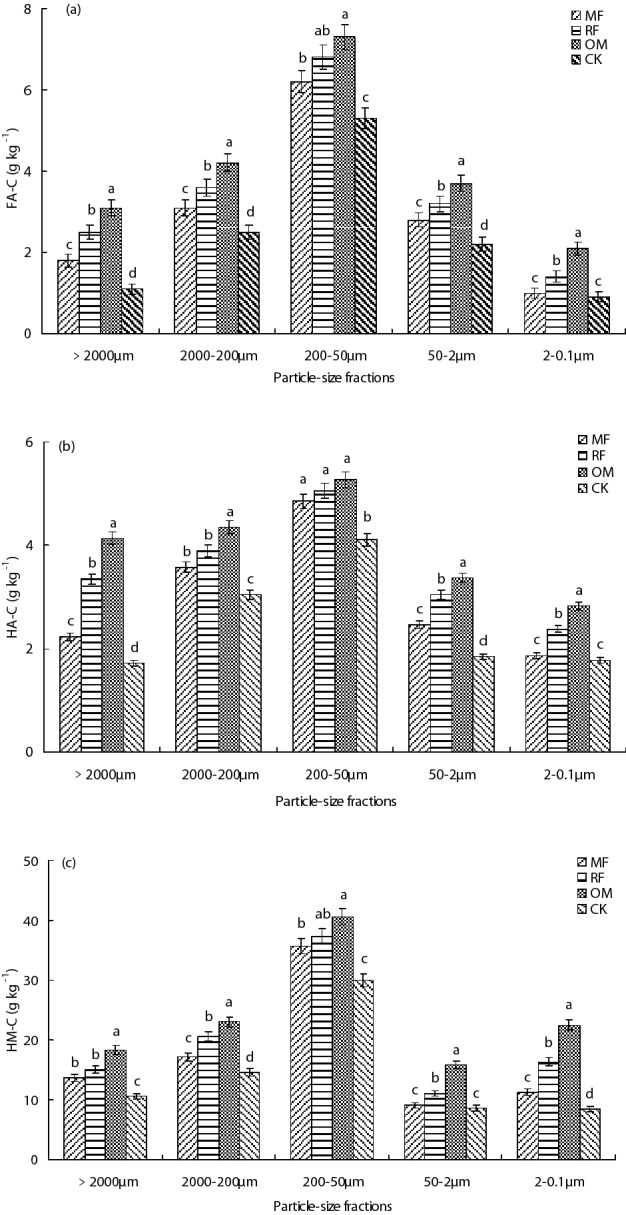
Carbon content of fulvic acid (FA), humic acid (HA) and humin (HM) from different particle-size fractions with different fertilizer treatments. *MF* chemical fertilizer alone, *RF* rice straw and chemical fertilizer, *OM* 30% organic manure and 70% chemical fertilizer, *CK* without fertilizer input as a control. **(a)** Carbon content of fulvic acid (FA-C), **(b)** carbon content of humic acid (HA-C), **(c)** carbon content of humin (HM-C). Different lowercase letters were indicated significant differences (*p* < 0.05) among different fertilizer treatments. Error bars represent standard error of the mean (n = 3).

In different particle-size fractions, the highest carbon (C) content of humus (FA, HA and HM) were found in 200–50 μm fraction, and the lower C content of humus were found in 50–0.1 μm fraction (Fig. 1a–c). Compared with MF treatment, the FA and HM C content in 2000–200 μm and 50–0.1 μm fractions with RF and OM treatments were significant (*p* < 0.05) higher (Fig. 1a,c), whereas there had not significant (*p* > 0.05) difference in HA C content in 200–50 μm fraction among MF, RF and OM treatments (Fig. 1b). The results indicated that FA, HA and HM C content in > 2000 μm and 2000–50 μm fractions with MF, RF and OM treatments were significant (*p* < 0.05) higher than that of CK treatment. Particle-size fractions and fertilizer treatments were significant (*p* < 0.05) influence on distribution of humus C components (FA, HA and HM), but their interaction influence on HA C content were not significant (*p* > 0.05) difference (Table 1).

**Table 1:** Two-way ANOVA analysis C content of soil humus, abundance of *cbhI* and *GH48* genes in the five soil particle-size fractions and fertilizer treatments.

	Particle-size fractions		Fertilizer		Particle-size fractions × fertilizer	
	*F*	*P*	*F*	*P*	*F*	*P*
FA-C	86.52	< 0.001	84.26	< 0.001	3.56	< 0.001
HA-C	91.67	< 0.001	61.58	< 0.001	**1.62**	0.135
HM-C	80.47	< 0.001	70.24	< 0.001	3.27	< 0.001
**Rice season**						
*cbhI* abundance	84.26	< 0.001	72.04	< 0.001	4.63	< 0.001
*GH48* abundance	12.35	0.146	14.17	0.185	**0.86**	0.362

The data in bold were indicated the parameter were not significant affected by soil particle-size fractions, fertilizer treatments or their interaction (*p* > 0.05).

*FA-C* carbon content of fulvic acid,* HA-C* carbon content of humic acid, *HM-C* carbon content of humin.

### CPMAS solid-state ^13^C NMR spectroscopy

The effects of different fertilizer treatments on humus C groups of FA and HA in five particle-size fractions were showed in Table 2. The results showed that alkyl C and *o*-alkyl C groups of FA in > 2000 μm fraction were significant higher (*p* < 0.05) than that of the other fractions (Table 2). However, carboxylic C of FA were most abundant in 50–2 μm and 2–0.1 μm fractions, which were resulted in significant higher ARO% of FA in these particle-size fractions. Aliphatic C of FA were most abundant in > 2000 μm and 200–50 μm fractions, which were resulted in significant higher AL% of FA in these particle-size fractions. There had no obvious significant (*p* > 0.05) difference in proportion of aromatic C group among the five particle-size fractions and fertilizer treatments. The alkyl/*o*-alkyl C of FA in 200–50 μm and 50–2 μm fractions were significant lower (*p* < 0.05) than that of the other particle-size fractions.

**Table 2:** Contribution of the different humus C type, aromaticity (ARO%), alphaticity (AL%) and alkyl/*o*-alkyl ratio were determined by using CPMAS ^13^C NMR of FA and HA in soil particle-size fractions under different fertilizer treatments.

Particle-size fractions	Treatments	0–45 ppm	45–95 ppm	95–165 ppm	165–200 ppm	ARO%	AL%	Alkyl/*o*-alkyl
		Alkyl C	*O*-alkyl C	Aromatic C	Carboxylic C			
**FA**								
>2000 μm	MF	26.7 ± 1.1c	30.7 ± 0.8a	22.6 ± 0.6a	17.5 ± 0.4ab	28.25 ± 0.84a	71.75 ± 2.02b	0.87 ± 0.02c
	RF	30.6 ± 0.9b	26.5 ± 0.8b	20.9 ± 0.6ab	18.3 ± 0.5a	26.79 ± 0.74ab	73.21 ± 2.14a	1.15 ± 0.04b
	OM	32.5 ± 0.8b	28.4 ± 0.7ab	20.4 ± 0.5b	18.7 ± 0.5a	25.09 ± 0.53b	74.91 ± 2.11a	1.14 ± 0.04b
	CK	38.6 ± 0.7a	18.5 ± 0.5c	21.5 ± 0.6ab	16.3 ± 0.3b	27.35 ± 0.76ab	72.65 ± 2.06ab	2.09 ± 0.05a
2000–200 μm	MF	18.5 ± 0.5b	14.2 ± 0.5b	23.1 ± 0.6a	40.5 ± 1.1a	41.40 ± 1.24a	58.60 ± 1.55b	1.30 ± 0.04a
	RF	20.1 ± 0.5ab	19.4 ± 0.6a	22.1 ± 0.5ab	36.7 ± 1.0ab	35.88 ± 1.16ab	64.12 ± 1.81ab	1.04 ± 0.03ab
	OM	20.6 ± 0.5a	20.3 ± 0.7a	22.6 ± 0.5ab	35.4 ± 0.9b	35.59 ± 1.10ab	64.41 ± 1.87ab	1.01 ± 0.02b
	CK	21.8 ± 0.6a	16.7 ± 0.5c	20.7 ± 0.4b	38.7 ± 1.1ab	34.97 ± 0.96b	65.03 ± 2.02a	1.31 ± 0.04a
200–50 μm	MF	28.6 ± 0.7ab	28.5 ± 0.7b	25.3 ± 0.7a	18.7 ± 0.6a	30.70 ± 1.05a	69.30 ± 1.67b	1.00 ± 0.03a
	RF	29.2 ± 0.8a	31.7 ± 0.9a	24.3 ± 0.6ab	17.6 ± 0.5ab	28.52 ± 0.95b	71.48 ± 1.78a	0.92 ± 0.02b
	OM	29.6 ± 0.8a	32.4 ± 0.9a	24.7 ± 0.6ab	17.1 ± 0.4b	28.49 ± 0.91b	71.51 ± 1.85a	0.91 ± 0.02b
	CK	27.4 ± 0.6b	30.3 ± 0.8ab	23.5 ± 0.5b	18.2 ± 0.6a	28.94 ± 0.97b	71.06 ± 1.93a	0.90 ± 0.02b
50–2 μm	MF	14.5 ± 0.5b	21.6 ± 0.6a	26.4 ± 0.8a	42.3 ± 0.9b	42.24 ± 1.15a	57.76 ± 1.43b	0.67 ± 0.01b
	RF	14.1 ± 0.4b	20.7 ± 0.5ab	25.1 ± 0.7ab	45.7 ± 1.1ab	41.90 ± 1.03b	58.10 ± 1.55ab	0.68 ± 0.01b
	OM	13.6 ± 0.3c	21.2 ± 0.6a	25.6 ± 0.7ab	46.8 ± 1.2ab	42.38 ± 1.18a	57.62 ± 1.47b	0.64 ± 0.01b
	CK	15.2 ± 0.5a	20.1 ± 0.4b	18.9 ± 0.6b	51.6 ± 1.3a	34.87 ± 0.96c	65.13 ± 1.68a	0.76 ± 0.02a
2–0.1 μm	MF	26.5 ± 0.7ab	12.1 ± 0.3c	31.6 ± 0.9a	40.7 ± 1.1ab	45.01 ± 1.32a	54.99 ± 1.35b	2.19 ± 0.08a
	RF	27.1 ± 0.7ab	16.6 ± 0.5a	29.4 ± 0.8ab	38.2 ± 1.1b	40.22 ± 1.17c	59.78 ± 1.63a	1.63 ± 0.05b
	OM	27.8 ± 0.8a	17.5 ± 0.5a	31.1 ± 0.9a	36.5 ± 1.0c	40.71 ± 1.13c	59.29 ± 1.58a	1.59 ± 0.05b
	CK	24.7 ± 0.6b	14.4 ± 0.4b	28.7 ± 0.7b	42.6 ± 1.1a	42.33 ± 1.24b	57.67 ± 1.46ab	1.72 ± 0.06ab
**HA**								
>2000 μm	MF	46.7 ± 1.2ab	33.4 ± 0.9a	25.4 ± 0.6b	12.7 ± 0.3ab	24.08 ± 0.45c	75.92 ± 1.91b	1.40 ± 0.04ab
	RF	44.5 ± 1.2ab	31.8 ± 0.7c	27.6 ± 0.6ab	13.1 ± 0.4a	26.56 ± 0.51b	73.44 ± 1.83c	1.40 ± 0.04ab
	OM	41.8 ± 1.1b	31.1 ± 0.6c	28.7 ± 0.7a	13.6 ± 0.4a	28.25 ± 0.56a	71.75 ± 1.76c	1.34 ± 0.02b
	CK	50.7 ± 1.3a	32.5 ± 0.8b	23.6 ± 0.5c	10.5 ± 0.2b	22.10 ± 0.37d	77.90 ± 1.97a	1.56 ± 0.05a
2000–200 μm	MF	34.7 ± 0.9a	29.3 ± 0.6c	25.3 ± 0.5b	13.1 ± 0.3ab	28.33 ± 0.73b	71.67 ± 1.75a	1.18 ± 0.05a
	RF	32.2 ± 0.8ab	30.6 ± 0.7ab	28.0 ± 0.7a	13.6 ± 0.3ab	30.84 ± 0.82ab	69.16 ± 1.64b	1.05 ± 0.04ab
	OM	31.5 ± 0.7b	31.2 ± 0.8a	28.6 ± 0.7a	14.2 ± 0.4a	31.33 ± 0.87a	68.67 ± 1.53b	1.01 ± 0.02b
	CK	33.6 ± 0.9ab	31.5 ± 0.8a	27.2 ± 0.6ab	11.4 ± 0.2b	29.47 ± 0.77b	70.53 ± 1.71ab	1.07 ± 0.04ab
200–50 μm	MF	23.5 ± 0.5c	28.7 ± 0.6ab	33.7 ± 0.8ab	15.6 ± 0.5a	39.23 ± 1.10a	60.77 ± 1.27b	0.82 ± 0.01b
	RF	25.2 ± 0.7a	29.6 ± 0.6ab	32.8 ± 0.8ab	14.6 ± 0.4ab	37.44 ± 1.04ab	62.56 ± 1.35ab	0.85 ± 0.02ab
	OM	25.8 ± 0.7a	30.2 ± 0.7a	31.7 ± 0.6b	15.1 ± 0.5a	36.15 ± 0.89b	63.85 ± 1.46a	0.85 ± 0.02ab
	CK	24.6 ± 0.6b	27.6 ± 0.5b	34.5 ± 0.9a	13.7 ± 0.3b	39.79 ± 1.16a	60.21 ± 1.20b	0.89 ± 0.03a
50–2 μm	MF	40.3 ± 1.1b	30.5 ± 0.7a	23.7 ± 0.4b	14.5 ± 0.5a	25.08 ± 0.52ab	74.92 ± 1.71ab	1.32 ± 0.03b
	RF	42.6 ± 1.2ab	28.8 ± 0.6ab	25.1 ± 0.5a	13.1 ± 0.4ab	26.01 ± 0.58a	73.99 ± 1.54b	1.48 ± 0.04ab
	OM	43.5 ± 1.2ab	29.6 ± 0.6ab	25.6 ± 0.5a	13.8 ± 0.4ab	25.94 ± 0.51ab	74.06 ± 1.67ab	1.47 ± 0.04ab
	CK	45.8 ± 1.3a	27.6 ± 0.5b	21.6 ± 0.3c	10.5 ± 0.2b	22.74 ± 0.42b	77.26 ± 1.78a	1.66 ± 0.05a
2–0.1 μm	MF	40.2 ± 1.0b	30.2 ± 0.8ab	26.1 ± 0.7a	12.7 ± 0.4ab	27.05 ± 0.76a	72.95 ± 1.47b	1.33 ± 0.03b
	RF	42.7 ± 1.1ab	31.2 ± 0.8ab	23.5 ± 0.6b	11.6 ± 0.3ab	24.13 ± 0.65ab	75.87 ± 1.64ab	1.37 ± 0.04ab
	OM	43.5 ± 1.3a	32.5 ± 0.9a	24.3 ± 0.6b	10.1 ± 0.2b	24.23 ± 0.61ab	75.77 ± 1.60ab	1.34 ± 0.03b
	CK	41.6 ± 1.1ab	28.8 ± 0.6b	21.7 ± 0.4c	14.9 ± 0.6a	23.56 ± 0.54b	76.44 ± 1.75a	1.44 ± 0.04a

Values followed by different lowercase letters within a column were indicated significant difference at *p* < 0.05.

*MF* chemical fertilizer alone, *RF* rice straw and chemical fertilizer, *OM* 30% organic manure and 70% chemical fertilizer, *CK* without fertilizer input as a control.

The results indicated that alkyl C of HA were the highest in the five particle-size fractions, and carboxylic C of HA were the lowest in the five particle-size fractions (Table 2). Meanwhile, this result showed that there had higher AL% and lower ARO% C of HA in different particle-size fractions. And the alkyl/*o*-alkyl C of HA were the lowest in 200–50 μm fraction and higher in 50–2 μm and 2–0.1 μm fractions with RF, MF and OM treatments.

### Abundance of *cbhI *and *GH48* genes

**Figure 2:**
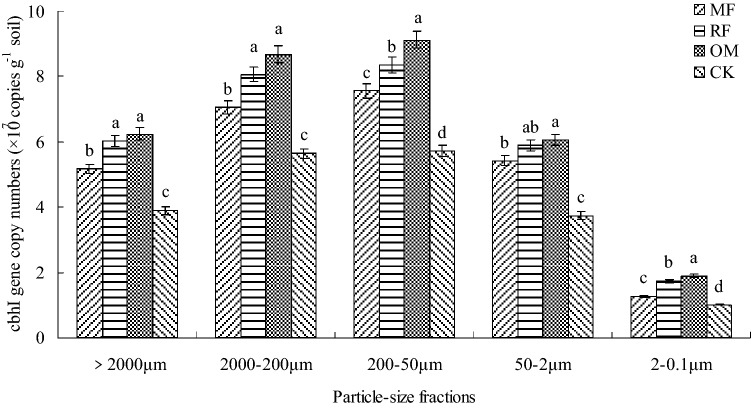
*cbhI* gene copy numbers in different particle-size fractions with long-term fertilizer treatments. *MF* chemical fertilizer alone, *RF* rice straw and chemical fertilizer, *OM* 30% organic manure and 70% chemical fertilizer, *CK* without fertilizer input as a control. Different lowercase letters were indicated significant differences (*p* < 0.05) among different fertilizer treatments. Error bars represent standard error of the mean (n = 3).

In different particle-size fractions, the range of abundance of *cbhI* gene with MF, RF, OM and CK treatments were 1.01–9.12 × 10^7^ copies g^−1^ soil (Fig. 2). The abundance of *cbhI* gene were significant impacted by particle-size fractions and fertilizer treatments individually and interactively (*P* < 0.001; Table 1). In different particle-size fractions, the abundance of *cbhI* gene with MF, RF and OM treatments were significant higher (*p* < 0.05) than that of CK treatment. Furthermore, the abundance of *cbhI* gene in different particle-size fractions with RF and OM treatments were significant higher (*p* < 0.05) than that of MF and CK treatments.

**Figure 3:**
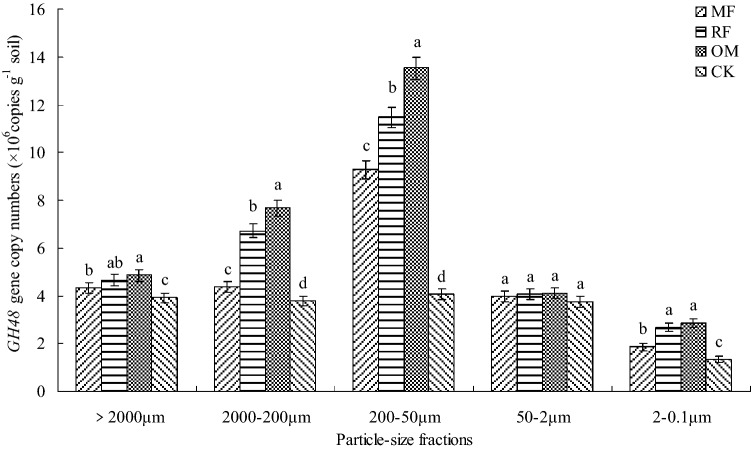
*GH48* gene copy numbers in different particle-size fractions with long-term fertilizer treatments. *MF* chemical fertilizer alone; *RF* rice straw and chemical fertilizer; *OM* 30% organic manure and 70% chemical fertilizer; *CK* without fertilizer input as a control. Different lowercase letters were indicated significant differences (*p* < 0.05) among different fertilizer treatments. Error bars represent standard error of the mean (n = 3).

In different particle-size fractions, the range of abundance of *GH48* gene with MF, RF, OM and CK treatments were 1.35–13.52 × 10^6^ copies g^−1^ soil, which were about ten times lower than that of abundance of *cbhI* gene (Fig. 3). This results indicated that abundance of *GH48* gene were obvious affected by particle-size fractions and fertilizer treatment individually, but there had no obvious significant (*p* > 0.05) affected by particle-size fractions and fertilizer treatment interactively (Table 1). The results showed that abundance of *GH48* gene in 200–50 μm fraction were significant higher (*p* < 0.05) than that of the other fractions. In different particle-size fractions, the abundance of *GH48* gene with MF, RF and OM treatments were significant higher (*p* < 0.05) than that of CK treatment. In > 2000 μm, 2000–200 μm and 200–50 μm fractions, the abundance of *GH48* gene with RF and OM treatments were significant higher (*p* < 0.05) than that of MF and CK treatments. However, there had no significant (*p* > 0.05) differences in abundance of *GH48* gene in 20–5 μm fraction between MF, RF, OM and CK treatments.

### Correlation analysis

There had significant (*p* < 0.01) positive correlation between soil β-glucosidase and β-cellobiohydrolase activity with abundance of *cbhI* and *GH48* genes (Table 3). Meanwhile, the abundance of *GH48* gene were significant (*p* < 0.01) correlated with soil properties (SOC, total N, and C/N) and soil humus C components (FA, HA and HM). And the abundance of *cbhI* gene were significant (*p* < 0.01) correlated with soil C/N, FA and HA C components (Table 3).

**Table 3:** Correlation coefficients between soil nutrient, humic C substances, enzyme activity and abundance of *cbhI* and *GH48* genes in different particle-size fractions under different fertilizer treatments.

	SOC	Total N	C/N	FA-C	HA-C	HM-C	β-glucosidase	β-cellobiohydrolase	*GH48*
*cbhI*	0.625*	ns	0.803**	0.785*	0.758*	0.547	0.782*	0.796*	0.807**
*GH48*	0.874**	0.506*	0.612*	0.891**	0.816**	0.806**	0.893**	0.851**	

*SOC* soil organic carbon, *FA-C* carbon content of fulvic acid, *HA-C* carbon content of humic acid, *HM-C* carbon content of humin, *ns* no significant relationship were detected.

^*^p < 0.05; ^**^p < 0.01.

Meanwhile, the results indicated that there had no significant (*p* > 0.05) relationship between alkyl C and aromatic C of FA with abundance of *cbhI* and *GH48* genes (Table 4). The C groups like o-alkyl C (*p* < 0.01) and AL% of FA (*p* < 0.05) were positively correlated with abundance of *cbhI* and *GH48* genes, however, ARO% were negatively (*p* < 0.05) correlated with abundance of *cbhI* and *GH48* genes. The results showed that both abundance of *cbhI* and *GH48* genes were negatively (*p* < 0.05) correlated with alkyl C, AL%, alkyl/*o*-alkyl C of HA, but abundance of *cbhI* and *GH48* genes were positively (*p* < 0.05) correlated with aromatic C and ARO% of HA (Table 4).

**Table 4:** Correlation coefficients between chemical compositions of C groups of FA and HA with abundance of *cbhI* and *GH48* genes in different particle-size fractions under different fertilizer treatments.

	Alkyl C	*O*-alkyl C	Aromatic C	Carboxylic C	ARO%	AL%	Alkyl/*O*-alkyl
**FA**							
*cbhI*	ns	0.702**	ns	ns	−0.527*	0.548*	−0.586*
*GH48*	ns	0.736**	ns	−0.514*	−0.536*	0.573*	Ns
**HA**							
*cbhI*	−0.735**	ns	0.568*	0.572*	0.608*	−0.607*	−0.705**
*GH48*	−0.612*	ns	0.545*	ns	0.614*	−0.593*	−0.613*

*ns* no significant relationship were detected.

^*^p < 0.05; ^**^p < 0.01.

## Discussion

Soil organic matter (SOM) is a complex and heterogeneous mixture of various substances, which has a continuum of decomposition and stabilization process in the soil^26^. Humus carbon (C) content was representing a large portion of natural organic matter distributed under different environmental condition, such as soil, sediment and water^27^. In the present study, this results indicated that humus C were occupied main component of soil organic carbon (SOC), which were consistent with previous reported^28^, who found that identified compounds accounted for 3.3% and 0.12% of humic acid C content, respectively. This results showed that higher alkyl C proportion and lower carboxylic C proportion of HA than that of FA in different particle-size fractions (Table 2), the reason may attribute to that HA were higher in molecular weight and contain less oxygen-containing functional groups when compared with FA^29^. In the previous study, the results indicated that ARO% of HA were two or three times higher than that of FA^30^, in the present study, the relatively higher ARO% of HA in 200–50 μm particle-size fraction and the relatively lower ARO% of HA in the other particle-size fractions when compared with FA, which probably due to that chemical structure of different humus component might depend on the heterogeneous environment that formed in different particle-size fractions^7^.

In the present study, the results showed that carboxylic C of FA in 50–2 μm and 2–0.1 μm fractions were higher than that of the other fractions (> 2000 μm, 2000–200 μm and 200–50 μm) (Table 2). Some results indicated the alkyl/*o*-alkyl C of soil FA were usually regarded as a sensitive index of SOM decomposition^31,32^, and the alkyl/*o*-alkyl C of soil FA were lower in 200–50 μm fraction but higher in 50–2 μm and 2–0.1 μm fractions in this study, which were suggested that there had higher SOM decomposition in 50–2 μm and 2–0.1 μm fractions^32^. Meanwhile, this results indicated that dominant C group of HA in 200–50 μm fraction were aromatic C and it were different from the other soil fractions that were dominated by alkyl C, thus resulting in the highest AL% and lowest ARO% in 200–50 μm fraction. Together with the lowest alkyl/*o*-alkyl C of HA in this fraction, these results further suggested that relatively lower humification degree in 200–50 μm fraction than that of the other fractions^33^. On the contrary, alkyl/*o*-alkyl C of HA in 50–2 μm and 2–0.1 μm fractions were higher than that of other particle-size fractions in this study, which were suggested that there were more stable organic matter and a possible accumulation of recalcitrant compound in these fractions^20,34^.

Cellulose-degrading enzyme plays an important role in decomposing soil microorganism, and the abundance of *cbhI* gene were usually regarded as a suitable marker for the cellulolytic fungi^14^. In this study, the abundance of *cbhI* gene were regarded as the fungal glycoside hydrolase family 7 from the *Basidiomycota* and *Ascomycota*^10^. Meanwhile, our results also indicated that abundance of *cbhI* gene in different particle-size fractions were changed under long-term application of fertilization condition, and the abundance of *cbhI* gene in different particle-size fractions were increased under combined application of crop residue and organic manure with chemical fertilizer condition (RF and OM treatments) (Fig. 2, Table 2), the reason maybe attributed to that there were more soil nutrient and C source with organic manure treatments, which were provide an appropriate soil environment, soil properties and nutrient for soil cellulolytic fungi multiplying^14,32^. However, some results indicated that abundance of cellulolytic gene were decreased under long-term application of mineral fertilizer condition^24^. Furthermore, the abundance of *cbhI* gene were significant related to the content of SOC and C/N ratio^15^. The lowest abundance of *cbhI* gene and higher total N content were showed in 2–0.1 μm particle-size fraction, thus there had no significant relationship between abundance of *cbhI* gene and total N content (Table 3). Soil β-cellobiohydrolase and β-glucosidase activities were significant correlated with abundance of *cbhI* gene (Table 3), suggested that change in abundance of these cellulolytic fungi of functional relevance in terms of C transformation in paddy ecosystem, consistent with the results of the previous studies^24,32^, who found that abundance of *cbhI* gene were relatively higher in fine sand but lowest in clay fraction, were generally increased with application of organic fertilizer treatment. However, there is still need further to study relationship between the other related rhizosphere soil enzyme activity and soil C transformation under long-term fertilization condition.

Multicellular actinomycetes and fungi were usually regarded as efficient cellulose decomposers, and actinobacteria plays an important role in SOM turnover, breakdown of recalcitrant molecules such as cellulose and polycyclic aromatic hydrocarbons^15,35^. In the present study, the results showed that abundance of *GH48* gene in different particle-size fractions were significant changed under long-term application of fertilization condition, were ten times lower than that abundance of *cbhI* gene (Figs. 2, 3). The reason maybe attributed to that qPCR primers did not included all actinobacterial *GH48* gene diversity from culture actinobacterial strains in the present experiment condition^15^. Therefore, the abundance of *GH48* gene in different particle-size fractions were lower, compared with abundance of *cbhI* gene. Meanwhile, our results indicated that abundance of *GH48* gene were highest in 200–50 μm fraction and lower in 50–2 μm and 2–0.1 μm fractions, and there had significant correlation between abundance of *GH48* with SOC, total N contents, C/N ratio and soil cellulase activity (Table 3). The reason maybe attributed to that high C/N ratio had negative influence on soil extracellular hydrolytic enzyme activity during litter decomposition, and soil enzyme activity synthesized by cellulolytic microbe in different particle-size fractions were increased with combined application of crop residue and organic manure with chemical fertilizer practice under lower soil C/N ratio condition, which were consistent with the results of previous studies^24,32^, who found that abundance of *GH48* gene were relatively higher in fine sand but lowest in clay fraction, were generally increased with application of organic fertilizer condition^36^.

In recent years, some results indicated that soil humification process is an important index of the change in amount and chemical structure of humus^32,37^. In the present study, the results showed that there had significant relationship between abundance of cellulolytic microbial with soil humus structural characteristic (Table 3). And the results also showed that C of HA were higher correlation with abundance of cellulolytic gene, suggested that C of HA were more active and sensitive to environment change than that of FA^32,38^. Meanwhile, there had some different relationship between C of FA and HA with abundance of cellulolytic gene (Table 4) for that naturally different characteristic of humus component^39^. In the previous study, these results indicated that o-alkyl C were generally considered as an easily indicator for the change of bio-decomposable organic component^21,40^. In this study, there had positively correlation between o-alkyl C of FA with abundance of cellulolytic gene and the negatively relationship between alkyl/o-alkyl C of HA with abundance of cellulolytic gene were confirmed that cellulose-degrading microbial community prefer the less humified fractions such as 200–50 μm fraction, rather than smaller fraction especially 2–0.1 μm fraction that contained more stable and recalcitrant substrate (Table 3, Figs. 2 and 3). The alkyl C, AL%, alkyl/o-alkyl C of HA were negatively (*p* < 0.05) correlated with both abundance of *cbhI* and *GH48* genes, and alkyl/o-alkyl C of FA had no correlation with abundance of *GH48* gene (Table 4), which were consistent with the results of previous studies^32,41^, who found that abundance of *cbhI* and *GH48* genes had negative correlation with alkyl/*O*-alkyl ratio C of HA, no correlation were detected between abundance of *GH48* gene with alkyl/*O*-alkyl ratio C of FA. However, due to the different distribution of functional microbial group in soil particle-size fractions mediating soil C transformation by using culture-independent method, further analysis were still needed to investigate the situ expression of glucoside hydrolase gene in rhizosphere soil under long-term fertilization condition.

## Conclusion

This result showed that distribution of humus carbon component (FA, HA and HM) were increased under long-term application of fertilization condition, and the FA, HA and HM C contents in > 2000 μm and 200–50 μm fractions with MF, RF and OM treatments were significant improved, compared without fertilizer input treatment. The results also indicated that alkyl C and oalkyl C groups of FA and HA in > 2000 μm fraction with different fertilizer treatments were higher than that of the other fractions, the order of relative abundance of different C groups of HA in different particle-size fractions were following alkyl C > *o*-alkyl C > aromatic C > carboxylic C. Compared without fertilizer input treatment, the abundance of *cbhI* and *GH48* genes in different particle-size fractions were increased under combined application of crop residue and organic manure with chemical fertilizer condition. There had close positive relationship between abundance of *cbhI* and *GH48* genes with chemical composition of FA and HA, which suggested that cellulolytic microorganism plays an important role in maintaining and changing chemical composition of FA and HA. However, further studies were necessary to investigate the role of soil fungi and actinobacteria in soil carbon cycling related to long-term fertilizer management.
